# Measuring Dyspnea and Perceived Exertion in Healthy Adults and with Respiratory Disease: New Pictorial Scales

**DOI:** 10.1186/s40798-015-0038-4

**Published:** 2016-01-07

**Authors:** Paolo T. Pianosi, Zhen Zhang, Paul Hernandez, Marianne Huebner

**Affiliations:** 1 Department of Pediatric and Adolescent Medicine, Mayo Clinic, 200 First St. SW, Rochester, MN 55905 USA; 2 Department of Statistics, University of Chicago, Eckhart 7 #5. 5734 S. University Avenue, Chicago, IL 60637 USA; 3 Department of Statistics and Probability, Michigan State University, 619 Red Cedar Rd, Rm C422 Wells Hall, East Lansing, MI 48824 USA; 4 Department of Medicine, Queen Elizabeth II Health Sciences Centre and Dalhousie University, 1796 Summer Street Halifax, Halifax, B3H 4G2 NS Canada

## Abstract

**Background:**

Dyspnea or perceived exertion during exercise is most commonly measured using Borg or visual analog scales, created for use in adults. In contrast, pictorial scales have been promoted for children due to skepticism concerning applicability of the said scales in pediatrics. We sought to validate our newly created, pictorial Dalhousie Dyspnea and Perceived Exertion Scales in adult populations and compare ratings with the Borg scale.

**Methods:**

Dyspnea and perceived exertion ratings obtained with both modified Borg CR-10 and Dalhousie scales during maximal cycle exercise were compared in 24 healthy adults and 17 with various pulmonary disorders. Scale ratings for perceived exertion were plotted against work while ratings for dyspnea were plotted against ventilation using previously developed alternative models to simple power law. Goodness of fit was determined by lowest root-mean-square error or by corrected Akaike information criterion.

**Results:**

Pictorial ratings of dyspnea and perceived exertion measured by both scale ratings rose as expected with increasing exercise intensity, and individual trajectories obtained by either scale were virtually superimposable in 90 % of subjects. In general, the lowest root-mean-square error or corrected Akaike information criterion was found with models which incorporated a time delay, defined as the fraction of maximum work or ventilation at which point a clear increase in ratings above resting level was reported.

**Conclusions:**

The Dalhousie Dyspnea and Exertion Scales offer an equally good alternative to the Borg scale for measuring dyspnea and perceived exertion in adults.

## Key Points

1. Dalhousie Dyspnea and Perceived Exertion Scales offer an alternative to Borg CR-10 scale in adults and were preferred by half our healthy subjects.
2. Most healthy subjects appear to have a lag or delay below which they report minimal changes in dyspnea or perceived exertion during incremental exercise, whereas most pulmonary patients do not, particularly for dyspnea.
3. Quadratic-delay model display improved fitting of observed trajectories of dyspnea perceived exertion during incremental, maximal exercise over simple power function.

## Background

Dyspnea is defined as a subjective experience of breathing discomfort that consists of qualitatively distinct sensations that vary in intensity [1]. Several scales have been employed to measure task-specific dyspnea: the most commonly employed is the Borg scale and modifications thereof [2–4]; another is a visual analog scale (VAS) [5–7]. Both VAS and Borg scales were developed and studied in adults, and the Borg scale specifically was originally conceived to rate the distinct but related sensation of perceived exertion. It has undergone a few iterations from its original description but remains the principal tool for quantitating these sensations during exercise. Borg scale ratings for dyspnea and leg effort conform to a stimulus–perceptual sensation relationship defined by [8] the following: *S = kI*^*a*^, where *S* is the magnitude of the particular sensation of interest (e.g., dyspnea), *I* the stimulus intensity, *k* a constant, and *a* the exponent, which lies somewhere between 1 and 2 in adults [2, 9].

We recently described mathematical modeling and perceived exertion ratings during incremental exercise to voluntary exhaustion in children and adolescents using the Borg scale [10]. We created an alternative scale for use in children and adolescents, Dalhousie pictorial scales [11], to measure dyspnea and perceived exertion during work requiring leg exercise such as cycling or running. We reported that the Dalhousie scales accurately track dyspnea and perceived exertion during a maximal exercise test in a pediatric population of individuals with and without respiratory disease [12]. Most recently, we demonstrated excellent correlations between ratings of dyspnea and ventilation, or perceived exertion and work intensity, during incremental exercise in children and adolescents [13]. We now report validation studies for our Dalhousie Dyspnea and Perceived Exertion Scales in adults, both healthy and with pulmonary disease, via three steps: (1) comparison with the current “gold standard,” i.e., Borg scale (concurrent validity); (2) Dalhousie pictorial ratings of dyspnea should rise with increasing ventilation and of perceived exertion should rise with increasing work (content validity); and (3) determination that rating trajectories conformed to a power function (internal validity). We studied adult subjects in order to open the door for potential use in adults with language or comprehension obstacles that could impair ability to dyspnea and perceived exertion using scales employing written and numeric cues.

## Methods

### Participants

Healthy adult subjects were recruited from hospital personnel or their friends at the IWK Health Centre in Halifax, Canada. Adult pulmonary patients underwent progressive exercise testing prior to beginning pulmonary rehabilitation at the Queen Elizabeth II Health Centre in Halifax, Canada. The Research Ethics Board of the IWK Health Centre approved this study, and subjects signed informed consent. All procedures were conducted in accordance with the Helsinki declaration of 1975.

### Procedures

Healthy adult subjects performed a maximal exercise test with step increments of either, 50, 100, or 150 kpm/min depending on a subject’s size and age on an electrically braked Collins ergometer. Increments were chosen to achieve test duration of 8–12 min, until voluntary, symptom-limited exhaustion (maximum work capacity, Wmax). Ventilation and gas exchange were measured on the same apparatus (CPX Plus, WE Collins, Braintree MA, USA). Adults with pulmonary disease performed incremental, symptom-limited exercise tests on a cardiopulmonary test system (Vmax Series 229, SensorMedics Corporation, Yorba Linda, CA) on an electronically braked cycle ergometer (Lode Corival 400, Groningen, Holland). The initial exercise work rate was set at unloaded pedaling and was increased using a ramp protocol at a rate of 100 or 150 kpm/min, depending upon the subjects’ self-reported functional exercise capacity. Tests were considered maximal in as much as subjects were encouraged to pedal to voluntary exhaustion or until the physician stopped the test for safety reasons.

### Symptom Measurement

**Fig. 1:**
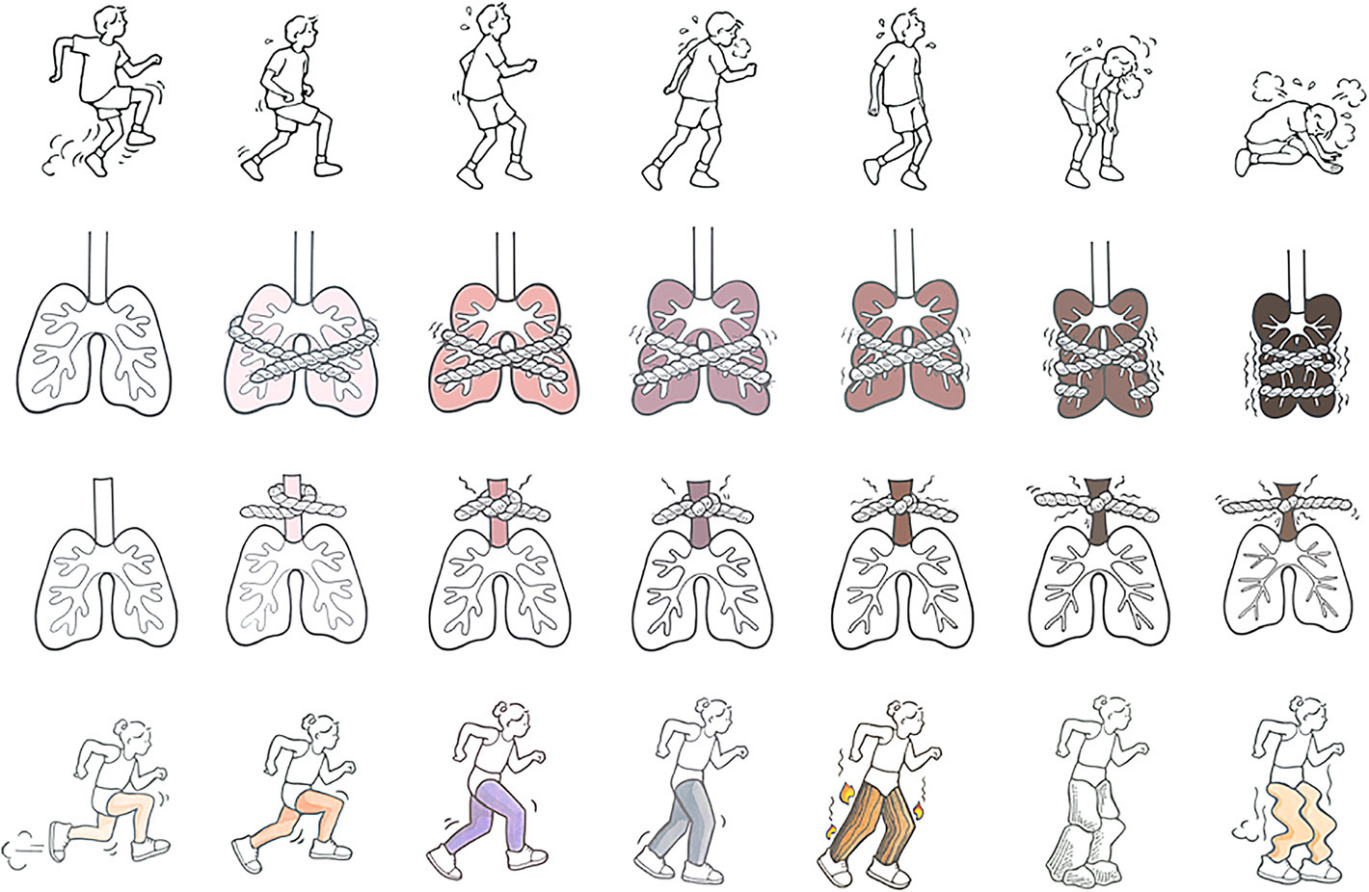
Dalhousie Dyspnea and Perceived Exertion Scales. Each scale depicts specific construct with severity increasing from left to right. The top row depicts breathing effort; the second set of pictures depict the construct of chest constriction or tightness; the third row throat narrowing; whereas the bottom row depicts the perceived exertion scale (for predominantly leg exercise)

Each scale was mounted in front of the participant. The Dalhousie Dyspnea and Perceived Exertion Scales consist of a sequence of seven pictures depicting three dyspnea contructs: chest tightness, throat closure, and breathing effort, plus an additional pictorial scale to depict leg exertion/fatigue (Fig. 1). The research assistant gave participants an explanation of the pictorial scales at the outset as follows:

The purpose of this test is to see how your breathing feels and how your legs feel during exercise. There is no right or wrong answer. The pictures in front of you show how your breathing might feel, from no difficulty at all, to the most difficulty you can imagine. You might feel this difficulty breathing in your chest or in your throat. Another scale simply asks you to tell us how hard it is to breathe – from nothing at all, to the hardest breathing imaginable. With the final set of pictures, tell us how your legs feel – from nothing at all, to the hardest imaginable. We will ask you the same using this other scale (pointing to the Borg CR-10).

Ratings using the pictorial scales at rest and during exercise were prompted by the questions:

“How does your breathing feel?” or “How do your legs feel?”

While ratings using the Borg scale were prompted by the questions:

“How hard is your breathing?” or “How tired are your legs?”

Ratings were recorded at each workload during the final 15–20 s of each work rate in the exercise tests on healthy controls but at rest and *every other* minute (alternating with blood pressure measurements) in pulmonary patients.

### Analysis

Baseline characteristics were summarized as mean ± SD or median (interquartile range [IQR]). We report and compare perceived exertion using the Borg CR-10 and Dalhousie pictorial (leg fatigue) ratings, as well as dyspnea ratings using the Borg CR-10 and Dalhousie pictorial (breathing effort) scales for simplicity. Concurrent validation was done with the premise that Borg’s scale is the gold standard. Dalhousie and Borg ratings were evaluated using canonical plots that compare actual ratings of one scale to the average ratings of the other scale at the same workload. Similar curves would indicate that ratings on one scale map well to the other scale. Internal validity was assessed by plotting dyspnea and perceived exertion ratings during increasing exercise intensity from measurements obtained using both Dalhousie and Borg scales and computing the mathematical stimulus–response relationship to determine the psychophysical function. For each individual subject, four models were defined with a delay or with a power term used to estimate a best fit mathematical model of leg fatigue or exertion vs work, and dyspnea—specifically, the breathing effort sub-scale—vs exercise ventilation (content validation). A delay was defined as %Wmax at which point a clear increase in ratings of leg symptom occurred or percentage of maximal ventilation at which dyspnea rating rose above resting level. A quadratic-delay model is defined as follows:

$$ \mathrm{S}=a+{b}_1{\left(I-d\right)}^{+}+{b}_2{\left({\left(I-d\right)}^{+}\right)}^2 $$

where *a* is an adjustment factor, *b*_1_ and *b*_2_ coefficients of the linear and quadratic term, respectively, and *d* denotes the delay. The coefficients of these models were estimated with a quasi-Newton algorithm. We calculated root-mean-square error (RMSE) and the Akaike information criterion (AIC) for model assessment, where the preferred model is the one with the lowest RMSE or AIC. These analyses were performed using R version 3.1.0: R Core Team (2014) (R Foundation for Statistical Computing, Vienna, Austria).

## Results

**Fig. 2:**
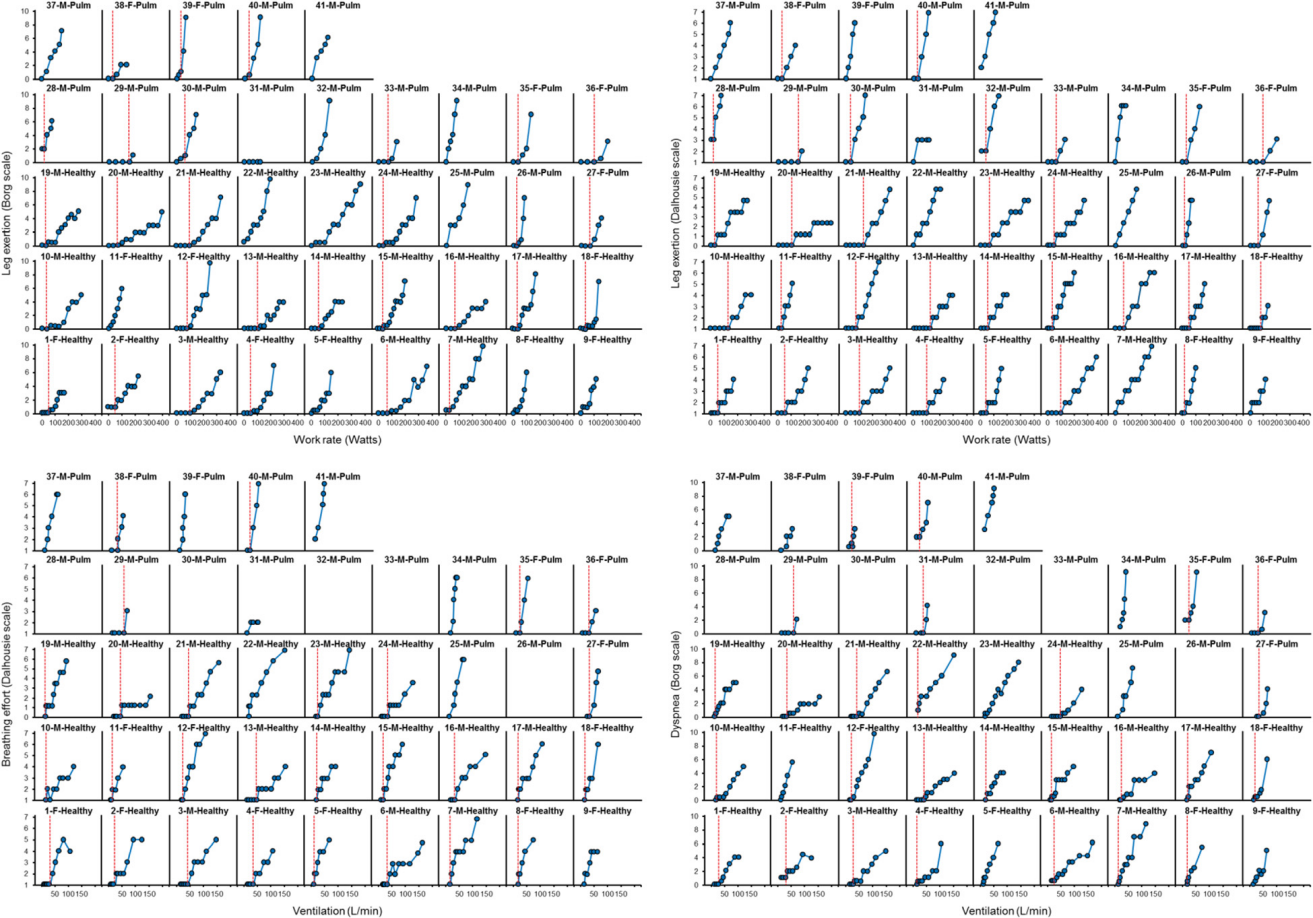
Line plots of dyspnea ratings obtained with breathing effort sub-scale of the Dalhousie scales and Borg dyspnea rating for each individual subject, plotted vs ventilation; and of perceived leg exertion obtained by Dalhousie and Borg scales vs work. *Dashed red vertical lines* indicate delay (“d” term in Table 3). One can see a general upward trend during incremental exercise but with marked inter-individual variability irrespective of diagnosis

Anthropometric, spirometric, and maximal exercise data on all subjects are shown in Table 1. Diagnoses in pulmonary patients were as follows: chronic obstructive pulmonary disease (COPD) 8, asthma 3, lung cancer 2, interstitial respiratory disease 2, pulmonary embolism 1, and bronchiectasis 1. Unfortunately, sub-maximal gas exchange data on five (Fig. 2) pulmonary subjects were lost due to computer malfunction (COPD 3, ILD 1, bronchiectasis 1). Adults with respiratory disease were older than healthy controls (*p* < .025). There was a 50:50 split among subjects when asked about scale preference following the test.

**Table 1:** Subject characteristics and maximal exercise data (means ± SD)

	Healthy controls	Pulmonary patients
M:F	15:9	12:5
Age (years)	37 ± 16	54 ± 15
Height (cm)	173.5 ± 10	171.6 ± 7
Weight (kg)	70.3 ± 13.5	91.6 ± 24.3
FVC (L)	5.33 ± 1.24	3.23 ± 0.64
FEV_1_ (L)	4.37 ± 0.94	2.09 ± 0.74
FEV_1_/FVC	0⋅82 ± 0⋅04	0⋅64 ± 0⋅16
$\overset{.}{V}{O}_{\mathsf{2}}$ (%predicted)^a^	105 ± 15	69 ± 13
$\overset{.}{V}{O}_{\mathsf{2}}$ (L· min^−1^)	2.72 ± 1.01	1.53 ± 0.31
${\overset{.}{V}}_E$ (L· min^−1^)	113 ± 34	53 ± 10
${\overset{.}{V}}_E$/35·FEV_1_	0⋅88 ± 0⋅28^a^	0⋅79 ± 0⋅24
Pulse (%predicted)	100 ± 6	80 ± 10

*FVC* forced vital capacity (L), *FEV*_1_ forced expired volume in first second (L), $\overset{.}{V}{O}_2$ oxygen uptake, ${\overset{.}{V}}_E$ ventilation

^a^peak $\overset{\acute{\mkern6mu}}{V}{O}_2$ = 0.046 (Ht) -0.021 (age) -0.62 (sex) -4.31 L/min peak pulse = 202 -0.72 (age), *after* [33]

**Fig. 3:**
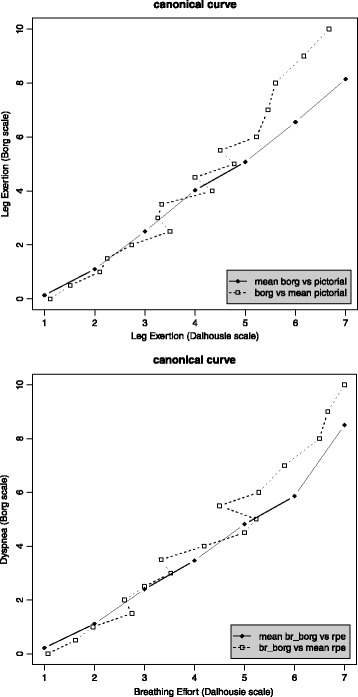
Canonical plots comparing ratings made by each scale. For example, when Borg perceived exertion rating was 10, Dalhousie leg fatigue ratings averaged just under 7 (*dashed line*). Alternately, when subjects rated leg fatigue with the sixth picture on the Dalhousie scales, averaged Borg rating fell between 6 and 7 (*solid line*). Similarly, when Borg dyspnea rating was 10, Dalhousie leg fatigue ratings averaged 7. Alternately, when subjects rated dyspnea at the fifth picture of the Dalhousie scales, averaged Borg rating was approximately 5

Results of model fitting these data are shown in Table 2. Inclusion of a delay term improved model fit [10]. Overall, a quadratic model with a delay term offered the best fit of any power function (*exp* = 2) from the data obtained based on lowest RMSE or AIC criteria. Median and quartiles of parameters for the quadratic-delay model are shown in Table 3. Curves for each individual subjects showing dyspnea rating using each scale vs ventilation and perceived exertion vs work are shown in Fig. 2. Ratings obtained with the throat and chest sub-scales of the pictorial scales mirrored those obtained using the perceived effort of breathing scale and are not shown. Two features differentiated pulmonary patients from healthy controls. There was clearly a steeper trajectory for dyspnea vs ventilation and for perceived exertion vs workload (parameter *b*_1_ in Table 3) for pulmonary patients than for healthy controls, regardless of which scale used; and there was a shorter delay between exercise onset and initial change from resting value (parameter d in Table 3) notwithstanding that healthy adults had more ratings (workloads) during exercise. The variation in the delay terms was large among individual subjects across all scales, but the largest differences were observed in Borg exertion ratings between healthy adults and patients with pulmonary disease: for Borg leg exertion scale vs %Wmax, the median delay was 64 % [IQR 42–100] vs 24 % [IQR 0–60] of their respective Wmax (Table 3). Canonical plots in Fig. 3 demonstrate that ratings by respective scales mirrored each other very well at light to moderate exercise then diverged slightly at peak exercise, as there was slightly more variability when ratings obtained by both scales during heavy exercise were compared. In spite of the fact that Dalhousie ratings ranged from 1 to 7, while the Borg ratings ranged from 0 to 10, ratings clearly differed at peak exercise in only two subjects: nos. 1 and 13 for dyspnea or nos. 14 and 16 for perceived leg exertion.

**Table 2:** Summary of model fitting

	Model	RMSE	AIC
Dalhousie legs vs W	Power	0.292	6.977
	Delay	0.245	−12.669
	Delay-power	0.247	6.454
	Delay-quadratic	0.196	−9.87
Borg leg fatigue vs W	Power	0.481	16.375
	Delay	0.418	−2.159
	Delay-power	0.395	15.809
	Delay-quadratic	0.344	−3.064
Dalhousie breathing effort vs ${\overset{.}{V}}_E$	Power	0.337	11.145
	Delay	0.324	3.976
	Delay-power	0.264	8.805
	Delay-quadratic	0.237	−2.157
Borg dyspnea vs ${\overset{.}{V}}_E$	Power	0.433	12.926
	Delay	0.352	−0.231
	Delay-power	0.295	9.901
	Delay-quadratic	0.269	−2.77

**Table 3:** Estimated model parameters for quadratic-delay model

	Model parameters	Healthy	Pulmonary dis.
Dalhousie legs vs %Wmax	*a*	1 (1.0, 1.05)	1 (1, 1.05)
	*b*1	0.02 (0.02, 0.02)	0.05 (0.03, 0.08)
	*b*2	0 (0, 0)	0 (0, 0)
	d	40.7 (27.7, 81.1)	26.7 (5.7, 41.5)
Borg leg fatigue vs %Wmax	*a*	0.18 (0.02, 0.36)	0.19 (0, 0.25)
	*b*1	0.02 (0.02, 0.03)	0.04 (0, 0.06)
	*b*2	0 (0, 0)	0 (0, 0)
	d	63.9 (41.6, 105)	24 (0, 60.4)
Dalhousie breathing effort vs %max ${\overset{.}{V}}_E$	*a*	1 (1, 1.15)	1 (1, 1.01)
	*b*1	0.06 (0.04, 0.11)	0.15 (0.08, 0.22)
	*b*2	0 (0, 0)	0 (0, 0)
	d	17.7 (14.9, 24.5)	23.7 (15.9, 29.8)
Borg dyspnea vs %max ${\overset{.}{V}}_E$	*a*	0.03 (0, 0.21)	0.02 (0, 1.68)
	*b*1	0.07 (0.04, 0.11)	0.11 (0, 0.22)
	*b*2	0 (0, 0)	0 (0, 0.01)
	d	20.2 (16.4, 30.0)	24.7 (15.9, 41.9)

Shown as median (1st, 3rd quartiles) for ratings perceived exertion and dyspnea, using Borg CR-10 scale or using Dalhousie scales. Model : S = *a* + *b*_1_(*I* − *d*)^+^ + *b*_2_((*I* − *d*)^+^)^2^

## Discussion

We demonstrated that dyspnea and perceived leg exertion ratings obtained using the Borg CR-10 scale closely tracked analogous ratings obtained using our Dalhousie scales in adult, rising more steeply in patients with pulmonary disease than in healthy controls but exponentially in both groups during incremental exercise. The coherence between measurements and similar trajectories obtained with either scale as exercise intensity grew implies that the Dalhousie pictorial scales can be used in adult populations with equal confidence and are arguably superior to the Borg scale in adults who have difficulty understanding the latter, e.g., patient 31.

We created the Dalhousie scales primarily as a tool to measure degree of dyspnea including the overall sense of breathing effort or discomfort and secondly perceived exertion with specific focus on the legs—the principal muscle groups involved in most ergometry systems. In his thesis, Borg stated the concept of overall perceived exertion could be regarded as a gestalt made up of perceptions from several important cues [3]. He later developed a category (C) scale with ratio (R) properties by adding numbers along his line at specific distances and adding descriptive adjectives to this non-linear numeric scale [4]. We submit that our pictures serve as the equivalent of, or replace, the descriptive categories for subjects who cannot grasp these sometimes-subtle, quantitative semantics. We further speculate that they embody the “qualitatively distinct sensations” [1] and the gestalt made up of perceptions from several important cues [3]. The larger variability in ratings obtained by the two scales seen during heavy exercise in canonical plots could simply be due to the additional gradations of sensation available on a CR-10 scale, but these extras matter little if a subject cannot distinguish “very strong” (7) from “extremely strong” (10). Killian argued that the Borg CR-10 scale adheres to principles of an absolute scale with ratio properties [14]. The Dalhousie Dyspnea and Perceived Exertions Scales function at least as interval scales with perceptual anchors (“from nothing at all, to the hardest breathing imaginable”), and our findings confirm their ratio properties as well. The novel finding of this study was that ratings of dyspnea and leg exertion derived from either Borg or Dalhousie pictorial scale conformed better to a trajectory that includes a delay term than to the expected simple power function.

Borg found it necessary to include one and sometimes two extra basic constants in his original equation (for the absolute threshold, or describing basic “noise”) [3, 4]. Borg and Kaijser ignored this delay term (eqtn.1 in their paper) [2], whereas Killian et al. noted thresholds in relative power output below which leg effort and dyspnea did not change appreciably from resting level [9]. This delay amounted to somewhere between 20 and 65 % (longest with Borg CR-10) of healthy subjects’ Wmax and accounts for some of the variability in dyspnea and perceived exertion ratings. It was markedly different between our healthy subjects and those with respiratory disease (Table 3) but we did not seek matched controls for purposes of this study since our aims were to compare scales, not healthy controls vs patients. In general, trajectories rose quite steeply in pulmonary patients from the outset, markedly different than growth function(s) in healthy controls. A model that accounts for variable delays *and* variability in rate of rise will be intuitively superior to a simple power function. The delay model that includes a linear and a quadratic term had the lowest RMSE and AIC, though coefficients for the quadratic terms were small. While ratio scales in general function well empirically, alternative modeling of the sensory–perceptual function, specifically incorporating a delay term, can reduce inter-subject variability in ratings.

Dyspnea is a complex sensation that relies on mechanical, chemical, and cortical inputs and incorporates numerous sensations, with a common theme of an imbalance or inappropriateness between neural output to, and mechanical output from, the respiratory pump [15–18]. Clearly, one becomes more aware of one’s breathing and the effort it requires as exercise becomes progressively harder or prolonged, but it does not become unpleasant or perceived as dyspnea so long as afferent signals from the respiratory apparatus remain in harmony with the efferent drive to the muscle(s) of breathing. This balance becomes upset by dynamic hyperinflation with exercise onset in patients with COPD or asthma in order to compensate for flow limitation [19–21] by low lung compliance coupled with flow limitation in ILD [22], whereas increased neural drive rather than mechanical ventilatory displacement appears to play a greater role in obesity [23]. Chemical stimuli arising from blood-gas abnormalities, particularly hypercapnia, both magnify and alter perceived dyspnea (and consequent affective response) [24], a common scenario in patients with respiratory disease. Patients with different disease processes tend to describe their sensation of dyspnea with typical descriptors [25–28]. Our pediatric focus group interviews during scale development differentiated the separate and distinguishable concepts of shortness of breath from increased breathing effort, prompting creation of the “breathing effort” sub-scale [2, 11]. There is now general agreement based on descriptor studies that perceptions of “work and effort,” “air hunger or unsatisfied breaths,” and “chest tightness” are separable qualities of dyspnea, and recent work suggests they are likely distinct [29]. In our study, all sub-scale (breathing effort, throat, or chest) ratings rose more or less in unison. We instructed subjects at the outset that their difficulty breathing might be perceived in the chest or throat, but we did not instruct them to choose one sensation or location over the other. “Descriptor” literature was a contemporaneous phenomenon during our scale development (1998) and work that elucidated mechanisms explaining separate but distinguishable types of dyspnea was only beginning when we conducted exercise tests. We demonstrated that dyspnea rose more steeply and to a greater extent in adults with pulmonary disease using our pictorial scales having receiving generic instructions. Future studies in this population can examine whether re-phrasing instructions to patients prompts them to select one scale over another to describe their sensory-perceptual experience. For example, it seems intuitive that an asthmatic experiencing bronchoconstriction may select the chest constriction scale, but will a COPD patient be drawn toward the throat narrowing scale to convey a sense of or unsatisfied inhalation (a common descriptor offered by adolescents with exercise-induced glottic obstruction) while both gravitate toward the breathing effort scale to describe exercise hyperpnea?

This issue may pose a limitation on applicability of the Dalhousie dyspnea and perceived exertion scales. One could argue that only the breathing effort and perceived leg exertion pictorial scales are all that are required, although one hopes that localization of the site of perceived difficulty might permit separation of patients with different disorders. No patients with cardiac disease were tested in our study, and utility of the Dalhousie scale in this population cannot be presumed. The pathophysiologic bases of dyspnea and perceived exertion scale in this population are arguably more complex [29, 30]. On the other hand, recent studies have considered the multidimensional aspect of dyspnea [30] that comprises three major aspects: a sensory-perceptual domain and a symptom impact both in physical and affective terms. Sensory-perceptual dimension includes ratings of dyspnea intensity and its quality, i.e., “what breathing feels like.” One might argue that there will be less precision differentiating perceived intensity levels denoted by solely by pictures without accompanying numbers. The pictorial nature of the Dalhousie scales implies de facto they are imbued with qualitative as well as quantitative information. It has been recently recognized that dyspnea, like pain, is a complex symptom with both sensory and affective domains, the latter thought to be processed in central limbic neural structures. Functional MRI has shown regions in the CNS where unpleasantness of perceived dyspnea is received, perceived, and processed [31, 32]. Furthermore, even verbal cues in the absence of any breathing load can evoke affective responses, which themselves are influenced by fatigue, anxiety, and somatic hypervigilance [32]. It is not unreasonable to presume that visual cues could do likewise, although we have no data for or against this notion. The Dalhousie pictorial scales were developed to quantify and discriminate qualitative aspects of the sensory perception of effort and difficulty associated with breathing in health and disease. Although the ratings are modulated by affective state in individual subjects, the pictorial scales were not developed to specifically assess the affective domain of dyspnea; further research would be necessary to develop pictorial scales to quantify the affective dimension of dyspnea. Finally, the perceived exertion scale is predicated on large-muscle, leg exercise—either treadmill or cycle ergometry. Arm cranking exercise is seldom performed in children (unless paraplegic), and thus, we convened no focus group to create the analogous arm-scale for this modality. Such would have to be created de novo if one wished to study perceived exertion with arm exercise. Such multiplicity of scales may render the entire pictorial concept unwieldy, but we believe this limitation will be more than compensated by the richness of information they yield.

## Conclusions

We conclude the Dalhousie Dyspnea and Perceived Exertion Scales yield comparable results to the Borg CR-10 scale in measuring dyspnea and perceived leg exertion during bicycle exercise in healthy adults and in adults with pulmonary disease. We already demonstrated feasibility and reproducibility of using the Dalhousie scales in a population of Italian children running on a treadmill in the hope that the pictorial nature of the scales would obviate need for verbal descriptors that might limit scale comprehension in subjects with limited literacy or understanding [13]. By the same reasoning, the Dalhousie scales can now be employed in exercise testing of adults in whom a clinician might have similar concerns. Our modeling to the stimulus–perceptual function suggests additional advantages of the Dalhousie Dyspnea and Perceived Exertion Scales. Specifically, efficacy of measures aimed at reducing exertional dyspnea can now be gaged either by reduction in dyspnea at the same absolute work, lengthening of the delay before dyspnea rises above resting level, or alteration in trajectory of dyspnea during progressive exercise, after an intervention.
